# Aberrant Mechanical Efficiency during Exercise Relates to Metabolic Health and Exercise Intolerance in Adolescents with Obesity

**DOI:** 10.3390/ijerph182010578

**Published:** 2021-10-09

**Authors:** Wouter M. A. Franssen, Guy Massa, Bert O. Eijnde, Paul Dendale, Dominique Hansen, Kenneth Verboven

**Affiliations:** 1 REVAL—Rehabilitation Research Center, Faculty of Rehabilitation Sciences, Hasselt University, 3590 Diepenbeek, Belgium; wouter.franssen@uhasselt.be (W.M.A.F.); dominique.hansen@uhasselt.be (D.H.); 2 BIOMED—Biomedical Research Center, Faculty of Medicine and Life Sciences, Hasselt University, 3590 Diepenbeek, Belgium; guy.massa@jessazh.be (G.M.); bert.opteijnde@uhasselt.be (B.O.E.); paul.dendale@jessazh.be (P.D.); 3 Department of Pediatrics, Jessa Hospital, 3500 Hasselt, Belgium; 4 Heart Centre Hasselt, Jessa Hospital, 3500 Hasselt, Belgium

**Keywords:** exercise tolerance, mechanical efficiency, physical fitness, metabolic risk factors, adolescents

## Abstract

Background. Mechanical efficiency (ME) might be an important parameter evaluating cardiometabolic health and the effectiveness of physical activity interventions in individuals with obesity. However, whether these cardiometabolic risk factors may relate to ME in adolescents with obesity is not known yet. Therefore, this study aims to compare the mechanical efficiency during maximal exercise testing between adolescents with obesity and lean adolescents, and to examine associations with exercise tolerance and metabolic health. Methods. Twenty-nine adolescents with obesity (BMI SDS: 2.11 ± 0.32, age: 13.4 ± 1.1 years, male/female: 15/14) and 29 lean (BMI SDS: −0.16 ± 0.84, age: 14.0 ± 1.5 years, male/female: 16/13) adolescents performed a maximal cardiopulmonary exercise test from which the net mechanical efficiency (ME_net_) and substrate oxidation (carbohydrates and lipids) were calculated. Indicators for peak performance were collected. Biochemistry (lipid profile, glycaemic control, inflammation, leptin) was studied in fasted blood samples. Regression analyses were applied to examine relations between ME_net_ and exercise tolerance or blood variables in the total group. Results. Peak work rate (WR_peak_), oxygen uptake (${\dot{V}O}_{2\mathrm{peak}}$)/WR_peak_, ME, and ME_net_ were significantly lower (*p* < 0.05) in adolescents with obesity compared to their lean counterparts (*p* < 0.05). Furthermore, a reduced ME_net_ was independently related to a lower WR_peak_ (SC β = 2.447; *p* < 0.001) and elevated carbohydrate oxidation during exercise (SC β = −0.497; *p* < 0.001), as well as to elevated blood low-density lipoprotein cholesterol (SC β = −0.275; *p* = 0.034) and fasting glucose (SC β = −0.256; *p* = 0.049) concentration. Conclusion. In adolescents with obesity, the mechanical efficiency is lowered during exercise and this relates to exercise intolerance and a worse metabolic health.

## 1. Introduction

The prevalence of obesity has increased considerably among adolescents over the past decades and has become one of the most significant health concerns worldwide [1]. Recent data indicate that the number of children and adolescents with obesity worldwide has increased up to 62 million in 2016 [1]. Obesity contributes to morphological and functional anomalies of adipocytes and adipose tissue, resulting in a pathophysiological process termed adiposopathy [2]. Due to adiposopathy, adolescents with obesity are at significantly greater risk to develop cardiovascular, metabolic, hepatic, respiratory, and orthopaedic disorders, compared to their non-obese peers [3]. As a result of these complications, childhood obesity alone results in a major economic burden, even if normalization of weight status is achieved during adulthood [4]. 

Cardiopulmonary exercise testing (CPET) provides valuable diagnostic and prognostic information of the integrative responses of the cardiovascular, pulmonary, and muscular system. During CPET, a lower peak cycling power output, oxygen uptake, and heart rate response to exercise are often observed in adolescents with obesity [5,6,7,8]. Next to these classical CPET parameters, mechanical efficiency (ME) has been examined as an important parameter evaluating muscular function and its relation to cardiometabolic health, as well as the effectiveness of physical activity interventions in adults with obesity [9,10]. ME refers to the ability of an individual to transfer the total metabolic energy costs into external work [11]. It has previously been shown that ME is reduced during steady state (submaximal) exercise in adults with obesity and, therefore, this population is limited in terms of physical activity, subsequently leading to a vicious cycle of physical inactivity and worse cardiometabolic health [12]. Although ME has been investigated in children and adolescents with obesity during submaximal exercise [7,8] it remains to be determined whether these differences could be observed during CPET, which is often performed as a comprehensive, time-efficient diagnostic test in clinical practice. However, for maximal exercise testing, current ME literature is controversial based on discrepancies in maximal effort indicators (e.g. peak heart rate and peak respiratory exchange ratio) [7]. In addition, these studies did not evaluate metabolic health which may be associated with ME. Of interest, physical activity intervention induced improvements in both ME and cardiometabolic health in sedentary obese adults [9], although no direct associations have been determined in current literature, especially in obese adolescents. Previous studies have shown that exercise capacity is negatively associated with metabolic risk factors such as insulin resistance in (adolescent) patients with obesity [13,14]. The relation between metabolic disease risk and cardiorespiratory fitness becomes even stronger in children with elevated adipose tissue mass [15]. Hence, it may be anticipated that these metabolic risk factors may relate to mechanical inefficiency. 

Therefore, the current study aimed (1) to compare ME during conventional CPET between adolescents with obesity and lean adolescents; and (2) to examine the associations between ME and determinants of metabolic health or exercise tolerance. It was hypothesized that ME is diminished during CPET, and that these alterations are associated with deviations in specific metabolic risk factors or exercise tolerance. If this hypothesis is confirmed, ME during CPET might be of clinical importance in adolescents with obesity as this can be a novel indicator for the presence or possible development of cardiometabolic complications among obese adolescents.

## 2. Materials and Methods

The study was carried out according to an observational, cross-sectional design, and was performed at the Jessa Hospital (Hasselt, Belgium) between November 2016 and July 2017, as described previously [5]. From midnight prior to a one-day hospitalization, all subjects refrained from consuming food, with the exception of water *ad libitum*, to prevent short-term metabolic effects on outcome parameters. After registration of general characteristics, such as anthropometry, body composition, Tanner stage, and blood pressure, venous blood samples were collected (in a fasted state) to examine participants’ cardiometabolic health. After this, one hour prior to CPET, a standardized meal (total energy: 296 kcal; composed of 3 g fats, 56 g carbohydrates and 9 g proteins) was consumed.

### 2.1. Participants

Adolescents with obesity were recruited from the pediatric clinic of Jessa Hospital (Hasselt, Belgium) and lean adolescents were recruited by means of paper advertisements at Jessa Hospital and Hasselt University (Hasselt, Belgium). Participants were between 11 and 17 years of age and free from any known chronic cardiovascular, renal, pulmonary, or orthopedic disease. Twenty-nine adolescents with obesity (BMI SDS: 2.11 ± 0.32, age: 13.4 ± 1.1 years, male/female: 15/14) and 29 lean adolescents (BMI SDS: −0.16 ± 0.84, age: 14.0 ± 1.5 years, male/female: 16/13) were included in this study based on the International Obesity Task Force criteria and body fat percentage (>95^th^ percentile) [16,17]. Body weight, BMI, BMI-SDS, waist circumference, hip circumference, waist-to-hip ratio, and the percentage of body fat (*p* < 0.001) were higher in subjects with obesity (Table 1 and Table S1). Both systolic (*p* < 0.001) and diastolic (*p* = 0.006) BP were increased in adolescents with obesity, compared to lean adolescents. All participants and their parents or legal guardians received oral and written information about the aim and protocol of the study and gave their written informed consent prior to participation. The study protocol was approved by the medical ethical committee of Jessa Hospital and Hasselt University (no. B243201214935) and was executed according to the Declaration of Helsinki (2013). The present study is registered at ClinicalTrials.gov with number NCT03516721.

### 2.2. Auxological Parameters and Blood Pressure

Body height was measured to the nearest 0.1 cm using a wall-mounted Harpenden stadiometer (ICD 250 DW, De Grood Metaaltechniek, Nijmegen, The Netherlands), with participants barefoot. Body weight (in underwear) was determined using a digital-balanced weighing scale to the nearest 0.1 kg (Seca 770, Hamburg, Germany). Body mass index (BMI) was calculated from weight and height measurements (weight/height^2^). The body height and BMI standard deviation scores (SDS) were calculated, as described by Cole et al., as: body height-SDS = [(body height/M)^L^ − 1]/[L *S] and BMI-SDS = [(BMI/M)^L^ – 1]/[L × S] [16,18]. Waist and hip circumferences were measured to the nearest 0.1 cm using a flexible metric measuring tape with participants barefoot (in underwear) in standing position. Waist circumference was measured at the midpoint between the lower rib margin and the top of the iliac crest. Hip circumference was measured at the widest circumference of the hip at the level of the greater trochanter. Waist-to-hip ratio was calculated by dividing waist circumference (cm) by hip circumference (cm). Body composition was evaluated using skinfold measurements. The thickness of the skinfolds were measured in triplicate at the left side of the body to the nearest 0.1 mm using an Harpenden skinfold caliper (Baty, West Sussex, UK), at the following sites: triceps, halfway between the acromion process and the olecranon process; biceps, at the same level as the triceps skinfold, directly above the center of the cubital fossa; subscapular, about 2 cm below the tip of the scapula, at an angle of 45° to the lateral side of the body; and suprailiac, about 2 cm above the iliac crest, in the axillary line [19]. The mean value of the triplicate measurements was used in the analysis. Skinfold measurements were performed by the same observer. The percentage of body fat was calculated using the equation reported by Slaughter et al. [20]. Pubertal status was assessed using Tanner’s scale according to observation by a pediatrician or the adolescents’ own opinion based on a figure. Blood pressure (BP, mm Hg) was measured in supine position using an electronic sphygmomanometer (Omron®, Omron Healthcare, IL, USA) after a resting period of five minutes. Mean arterial pressure (MAP, mm Hg) was calculated as MAP = systolic BP + (2 * diastolic BP)/3. 

### 2.3. Biochemical Analyses

After antecubital catheter placement, venous blood samples were taken for the measurement of biochemical blood parameters. Plasma glucose (mg/dL), uric acid (mg/dL), lipid profile (blood total cholesterol (mg/dL), high-density lipoprotein (HDL, mg/dL) cholesterol, low-density lipoprotein (LDL, mg/dL) cholesterol and triglyceride concentration [mg/dL]), c-reactive protein (CRP, mg/dL) and serum insulin concentrations (mU/L) were automatically assessed using Roche Cobas 8000 (Roche Diagnostics International Ltd, Rotkreuz, Switzerland). Blood glycated haemoglobin (HbA1c, %) concentration was measured using ion exchange chromatography (Menarini HA-8180 HbA1c auto-analyser, Menarini Diagnostics, Diegem, Belgium). Serum leptin concentration (µg/l) was measured using radioimmunoassay (RIA; Linco Research Inc., Saint Louis, MI, USA). Whole-body insulin resistance was estimated using the homeostatic model assessment for insulin resistance (HOMA-IR) and calculated as fasting glucose concentration (mg/dL) * fasting insulin concentration (µU/mL)/405 [21]. 

### 2.4. Cardiopulmonary Exercise Testing (CPET)

CPET was performed up to volitional exhaustion using an electronically braked cycle ergometer (eBike, GE Medical systems, Milwaukee, WI, USA), controlled by the Cardiosoft electrocardiography software (Cardiosoft 6.6, GE Medical systems, Milwaukee, WI, USA). At the beginning of each test day, a gas and volume calibration was performed according to manufacturer’s instructions. During the test, environmental temperature was kept stable at 19–21 °C. The exercise test (ramp protocol) included a one-minute pre-exercise resting period, a one-minute warm-up cycling (unloaded exercise) period, and an incremental exercise cycling phase with an initial work rate of 40 W with an increasing work rate of 20 W per minute until exhaustion or other symptoms limiting the test. During warm-up cycling and incremental exercise, a cycling frequency of 60 to 70 revolutions per minute (rpm) had to be maintained. The test was ended when the individuals failed to maintain a pedal frequency of at least 60 rpm. All subjects were verbally encouraged during exercise testing to achieve maximal effort, based on a respiratory gas exchange ratio (RER) of ≥1.10, and subjective opinion of an experienced tester who confirmed whether a maximal exercise test was executed, based on subjective features, such as dyspnea, sweating, facial flushing, clear unwillingness to continue, and a sustained drop in the participants pedaling frequency despite verbal encouragement, as described by Bongers et al. [22]. Due to the potential presence of chronotropic incompetence in adolescents with obesity, the heart rate at peak exercise (HR_peak_, bpm) was not used as a criterion for maximal exercise effort [23]. After cessation of exercise, work rate was set at 45 W at which subjects cycled during two minutes for active recovery with a cycling frequency of 50 rpm.

Continuous pulmonary gas exchange analysis (Jaeger MasterScreen CPX Metabolic Cart, CareFusion Germany GmbH, Hoechberg, Germany) was used to measure oxygen uptake (${\dot{V}O}_{2}$, mL/min), carbon dioxide output (${\dot{V}\mathrm{CO}}_{2}$, mL/min), minute ventilation ($\dot{V}$E, l/min), equivalents for oxygen uptake ($\dot{V}$E/${\dot{V}O}_{2}$) and carbon dioxide output ($\dot{V}$E/${\dot{V}\mathrm{CO}}_{2}$), tidal volume (Vt, l), breathing frequency (f, breaths per minute) and RER, and collected breath-by-breath with averaging every ten seconds. First ventilatory threshold (VT1) was determined using the V-slope method and was expressed in mL/min ${\dot{V}O}_{2}$ [24]. The second ventilatory threshold (VT2) was determined, using the $\dot{V}$E vs. ${\dot{V}\mathrm{CO}}_{2}$ plot, on the point where $\dot{V}$E increases out of proportion to ${\dot{V}\mathrm{CO}}_{2}$ and expressed in mL/min ${\dot{V}O}_{2}$ [25]. In addition, peak work rate (WR_peak_, Watt) was reported. The mechanical efficiency (ME) of cycling was determined, as described by Garby et al., and calculated as WR (Watt)/total energy consumption (mL/min), whereby the energy expenditure was calculated as [(4.94 × RER + 16.04) × (${\dot{V}O}_{2}$, in mL/min)/60] × 100 [26]. The net ME (ME_net_) was calculated from the ratio of work performed to the rate of energy expenditure above rest as described by Lafortuna et al. [12]. Using a 12-lead ECG device (KISS™ Multilead, GE Medical systems, Freiburg, Germany) HR was monitored and averaged every ten seconds. Substrate oxidation (g/min) was calculated based on ${\dot{V}O}_{2}$ and ${\dot{V}\mathrm{CO}}_{2}$, as described by Frayn et al. [27].

### 2.5. Statistical Analysis

Statistical analysis was performed by IBM SPSS^®^ version 24.0 (IBM SPSS Statistics for Windows, Chicago, IL, USA). Data were expressed as mean ± standard deviation (SD). Shapiro–Wilk tests were used to test normality of the data (*p* < 0.05). Comparisons between groups were tested using the chi-square test for categorical variables. Differences between continuous variables were assessed using independent sample T-tests for normally distributed data and Mann–Whitney U-tests for abnormally distributed data. A two-way repeated-measures ANOVA was used to assess whether there were differences in ME between adolescent with obesity and lean adolescents: an interaction effect was evaluated, where group (adolescents with obesity vs. lean adolescents) was a between-subjects factor, and time (percentage of ${\dot{V}O}_{2\mathrm{peak}}$) was a within-subjects factor. A post-hoc analysis (Bonferroni post-hoc comparison test) was performed when the between-subjects factor was statistically significant. Multivariate linear regression analysis was applied to examine relations between altered ME and metabolic health parameters or CPET parameters in both groups. In these regression analyses, corrections for age, sex, and the Tanner stage were made. Variables with a beta-coefficient <0.1 were left out of consideration. A *p*-value <0.05 (2-tailed) was considered statistically significant. 

The sample size calculation was performed using GPower v. 3.1 (Düsseldorf, Germany) and based on a previous study from Marinus et al. that showed a reduced WR_peak_ (effect size d: 0.93) in adolescents with obesity [28]. Based on a statistical power >0.8 and a two-sided alpha of 0.05, it was calculated that a sample size of at least 26 individuals with obesity and 26 healthy controls had to be included in the present study. Taking into account a drop-out rate of 10%, the number of participants to include in this study was at least 29 lean and 29 adolescents with obesity, resulting in a final sample size of 58 subjects. 

## 3. Results

### 3.1. Biochemical Parameters 

Blood HDL cholesterol concentration was lower (*p* < 0.05), whereas blood uric acid, CRP, LDL cholesterol, triglycerides, triglyceride-to-HDL cholesterol ratio, glucose, insulin, and leptin concentrations were all higher (*p* < 0.05) in adolescents with obesity (Table 2 and Table S2). Whole-body insulin resistance reflected by HOMA-IR was elevated (*p* < 0.001) in adolescents with obesity compared to lean adolescents.

### 3.2. Exercise Tolerance and Mechanical Efficiency 

At rest, $\dot{V}$O_2rest_ (mL/min: *p* = 0.036), $\dot{V}$CO_2rest_(*p* = 0.017), HR_rest_ (*p* < 0.001), and carbohydrate oxidation *p* < 0.001) were found to significantly increase in adolescents with obesity compared to lean adolescents. In addition, a lower $\dot{V}$O_2rest_ (mL/min/kg: *p* < 0.001) was found in adolescents with obesity. Furthermore, a lower lipid oxidation (*p* = 0.033) was observed in adolescents with obesity (Table S3).

**Figure 1:**
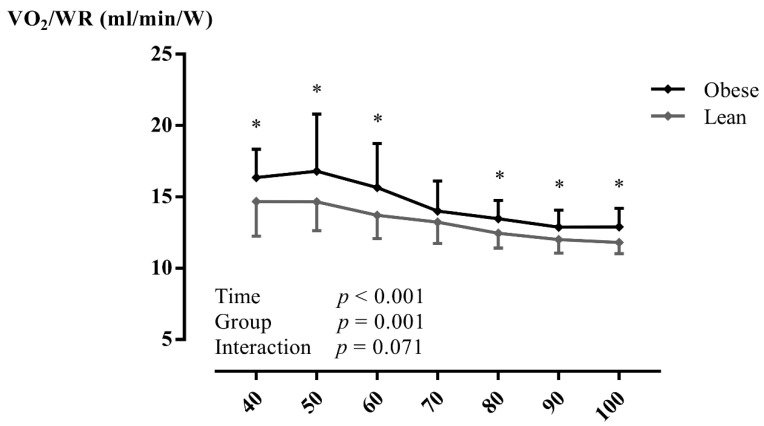
The oxygen uptake per work rate in relation to percentage of peak oxygen uptake in lean subjects and subjects with obesity during maximal exercise testing. Data are presented as mean ± SD. A two-way repeated-measures ANOVA (with post-hoc Bonferroni correction) was used to assess whether there were differences in mechanical efficiency during exercise testing between adolescents with obesity and lean adolescents. Abbreviations: ${\dot{V}O}_{2}$/WR: oxygen uptake per work rate, W: Watt. * *p* < 0.05 between groups.

**Figure 2:**
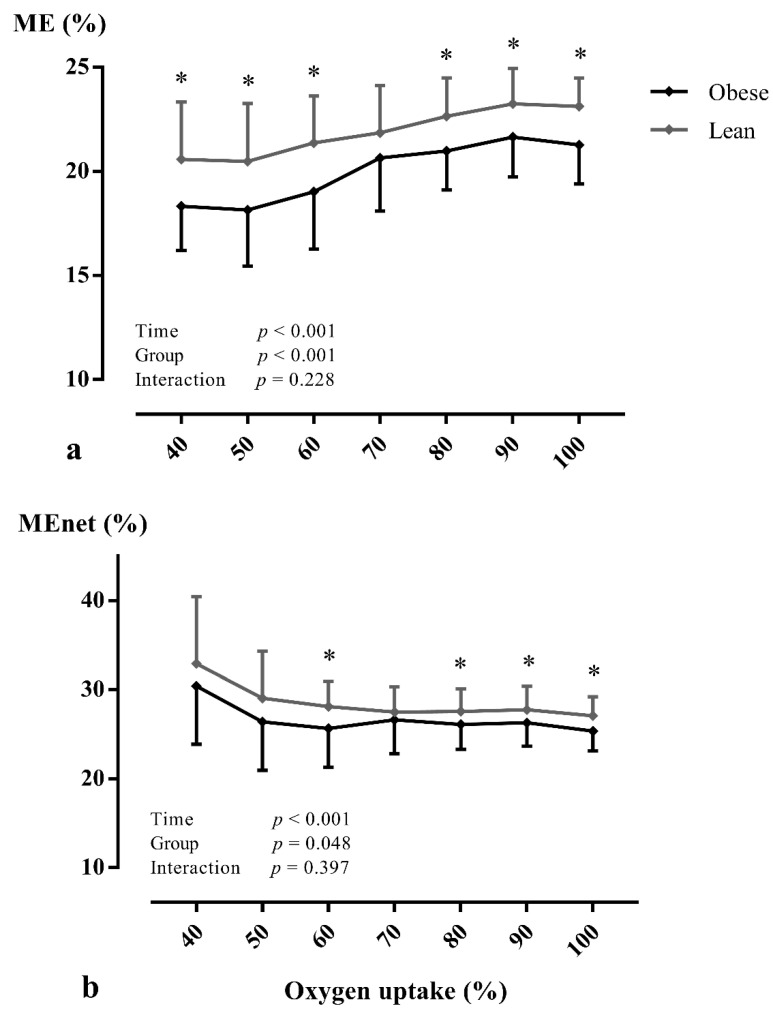
The mechanical efficiency (**a**) and net mechanical efficiency (**b**) in relation to percentage of peak oxygen uptake in lean subjects and subjects with obesity during maximal exercise testing. Data are presented as mean ± SD. A two-way repeated-measures ANOVA (with post-hoc Bonferroni correction) was used to assess whether there were differences in mechanical efficiency during exercise testing between adolescents with obesity and lean adolescents. Abbreviations: ME: mechanical efficiency, MEnet: net mechanical efficiency. * *p* < 0.05 between groups.

At peak exercise, a significantly reduced ${\dot{V}O}_{2\mathrm{peak}}$ (mL/min/kg: *p* < 0.001), WR_peak_ (*p* = 0.010), ${\dot{V}O}_{2\mathrm{peak}}$/WR_peak_, ME (*p* < 0.001), and ME_net_ (*p* = 0.005) were found in adolescents with obesity (Table 3). During CPET, ${\dot{V}O}_{2\mathrm{peak}}$/WR_peak_ (Figure 1 and Figure S1) was significantly higher (within-subjects: *p* < 0.001; between between-subjects: *p* = 0.001) and ME (Figure 2a and Figure S2) was significantly lower (within-subjects: *p* < 0.001; between between-subjects: *p* < 0.001) in adolescents with obesity, irrespective of gender. In addition, ME_net_ (Figure 2b) was significantly reduced (within-subjects: *p* < 0.001; between between-subjects: *p* = 0.048) in adolescents with obesity at low-, moderate-, and vigorous-intense exercise. In addition, similar results were found at VT1 and VT2 (Table S3). However, no interaction effects (adolescents with obesity vs. lean adolescents as a between-subjects factor and percentage of ${\dot{V}O}_{2\mathrm{peak}}$ as a within-subjects factor) were found. No other CPET parameters differed between groups (*p* > 0.05).

### 3.3. Associations between Mechanical Efficiency and Exercise Tolerance or Metabolic Health 

A higher ME_net_ at peak exercise was independently related (model r^2^ = 0.774; *p* < 0.001) to a higher WR_peak_ (SC β = 2.447; *p* < 0.001) and diminished CHO (SC β = −0.497; *p* < 0.001). With regard to metabolic health, a lowered ME_net_ was significantly and independently associated (model r^2^ = 0.134; *p* = 0.021) with elevated blood LDL cholesterol (SC β = −0.275; *p* = 0.034) and glucose (SC β = −0.256; *p* = 0.049) concentrations.

## 4. Discussion

In the present study, a decreased ME_net_ was present in adolescents with obesity. This is independently associated with a lowered WR_peak_, elevated blood LDL cholesterol, and fasting glucose concentrations. Data from the present study thus seem to verify that obesity, even at young age, is related to mechanical inefficiency during exercise, which relates to exercise intolerance and a worse metabolic health. 

At rest, an increase in ${\dot{V}O}_{2}$, ${\dot{V}\mathrm{CO}}_{2}$, and HR was found in adolescents with obesity. It has been shown that energy expenditure in children and adolescents with obesity increased at rest, possibly due to their increased fat free mass and sympathetic drive [28,29]. In addition, WR_peak_ was significantly reduced by ~15% in adolescents with obesity, indicating that exercise intolerance is prevalent in this population. In a recent meta-analysis, it remained uncertain whether WR_peak_ would be reduced consistently in adolescents with obesity, although two out of three studies reported a reduced WR_peak_ [6]. This reduced WR_peak_ was observed in presence of a maintained ${\dot{V}O}_{2\mathrm{peak}}$, thus indicating that the total oxygen uptake and transport is not the limiting factor during exercise in this population, but how oxygen is used in muscular metabolism.

A reduction in WR_peak_ may indeed result from exercise inefficiency of the skeletal muscles, as described previously in adults with obesity [30]. In line with these findings, data from the present study show significantly that ME (by 8%) and ME_net_ (by 7%) decreased, and that ${\dot{V}O}_{2\mathrm{peak}}$/WR_peak_ (by 10%) increased in adolescents with obesity, and an independent relation between a reduced ME_net_ and WRpeak (SC β = 2.447; *p* < 0.001). A reduced skeletal muscle work efficiency is hypothesized to be caused by metabolic muscle dysfunction [31] and/or a reduced mitochondrial content [32]. Moreover, the endocrine axes, including thyroid hormones and leptin, may play a role in the development of a decreased skeletal muscle ME [33,34]. These hormones affect the relative proportions of muscle fiber types [35], skeletal muscle glycolytic capacity, and alterations in the proportions of isoforms of the heavy chain of myosin (MHC) [33]. In addition, studies have shown that the skeletal muscles of adults with obesity are comprised of a lower proportion of oxidative type I skeletal muscle fibers [36]. This would lead to an attenuated skeletal muscle oxidative metabolism in individuals with obesity [31]. Although the relationship between muscle fiber type and obesity in adolescents has not been investigated, limited evidence of impaired exercise fat oxidation in pubertal boys with obesity was shown by Zunquin et al. [37]. Another explanation for deficits in skeletal muscle oxidative metabolism in individuals with obesity is a reduction in key enzymes associated with the oxidation of fats, leading to a reduced capacity for fatty acid oxidation in skeletal muscles [38]. Our results are in line with these previous findings, since significantly reduced lipid oxidation and increased carbohydrate oxidation were both noticed at rest in adolescents with obesity. In addition, this probably explains the independent association between a lower ME_net_ and a higher carbohydrate oxidation during CPET. 

A lowered ME_net_ is independently associated with elevated blood LDL cholesterol (SC β = −0.275; *p* = 0.034) and fasting glucose (SC β = −0.256; *p* = 0.049) concentrations in adolescents with obesity. It thus follows that obese adolescents with mechanical inefficiency are more likely to display a disturbed blood lipid profile and higher fasting glucose levels. It has been shown that both high blood LDL cholesterol levels and fasting glucose concentration are associated with insulin resistance [39,40]. It may be hypothesized that insulin resistance relates to skeletal muscle mitochondrial dysfunction [41], and hence a reduced ME_net_ and WR_peak_. 

Data from the present study indicate that mechanical efficiency during exercise may be an important clinical parameter that deserves greater attention in adolescents with obesity. Moreover, since mechanical efficiency is linked to metabolic health and exercise tolerance, it may be hypothesized that exercise intervention/therapy should be offered to every adolescent with obesity with the aim to normalize the mechanical efficiency during exercise [9]. As a result, it is expected that a higher ME will improve metabolic health. 

### Limitations and Future Directions

This is the first study that relates mechanical inefficiency during CPET to a worse metabolic health and exercise intolerance in adolescents with obesity. Despite these innovative findings, there are several limitations of this study. The primary limitation of this study is the cross-sectional design which does not allow the examination of causality. Therefore, data obtained from prospective studies with training and detraining effects are recommended to confirm our findings. We classified groups with obesity based on BMI without using a valid and reliable measure of body composition. In future research, it is recommended to characterize different subjects by body composition measured using dual-energy x-ray absorptiometry or MRI.

## 5. Conclusions

This study demonstrates that, in adolescents with obesity, ME during exercise is significantly impaired, which relates to exercise intolerance and elevated blood LDL cholesterol and glucose concentrations. Therefore, ME could be used as an important parameter evaluating (cardio)metabolic health.

## Figures and Tables

**Table 1:** Subject characteristics of obese and lean individuals.

General Features	Obese (*n* = 29)	Lean (*n* = 29)	*p*-Value
Age (years)	013.4 ± 1.10	014.0 ± 1.50	0.078
Sex			0.792
Male (*n*)	15	16	
Female (*n*)	14	13	
Body weight (kg)	088.0 ± 15.3	054.7 ± 10.8	<0.001
Body height (cm)	166.7 ± 8.70	166.8 ± 8.90	0.949
Body height-SDS	0.82 ± 0.99	0.68 ± 1.00	0.595
BMI (kg/m^2^)	031.6 ± 4.20	019.5 ± 2.40	<0.001
BMI-SDS	2.11 ± 0.32	−0.16 ± 0.84	<0.001
Waist circumference (cm)	103.0 ± 12.8	067.4 ± 6.20	<0.001
Hip circumference (cm)	104.1 ± 8.20	078.7 ± 8.30	<0.001
Waist-to-hip ratio	00.99 ± 0.07	00.86 ± 0.11	<0.001
Body fat (%)	047.6 ± 10.7	18.0 ± 5.7	<0.001
Body fat (kg)	42.7 ± 15.5	10.1 ± 4.3	<0.001
Fat-free mass (kg)	45.3 ± 8.7	44.6 ± 8.4	0.766
Systolic BP (mmHg)	125 ± 11	114 ± 10	<0.001
Diastolic BP (mmHg)	76 ± 10	70 ± 7	0.006
Mean arterial pressure (mmHg)	92 ± 8	85 ± 7	<0.001
Development stage			0.271
Tanner stage 1 (*n*)	3	2	
Tanner stage 2 (*n*)	0	2	
Tanner stage 3 (*n*)	8	4	
Tanner stage 4 (*n*)	3	7	
Tanner stage 5 (*n*)	15	14	

Data are expressed as mean ± SD. Abbreviations: SDS: standard deviation score, BMI: body mass index, BP: blood pressure. Comparisons between groups were tested using the chi-square test for categorical variables. Comparisons between groups were performed using the independent-samples t-test or Mann–Whitney U test for continuous variables.

**Table 2:** Biochemical and hormonal parameters in obese and lean adolescents.

	Obese (*n* = 29)	Lean (*n* = 29)	*p*-Value
Cardiovascular risk factors			
C-reactive protein (mg/l)	04.2 ± 6.9	00.3 ± 0.8	<0.001
Total cholesterol (mg/dL)	158 ± 33	150 ± 24	0.308
LDL cholesterol (mg/dL)	094 ± 27	075 ± 22	0.004
HDL cholesterol (mg/dL)	045 ± 11	061 ± 12	<0.001
Triglycerides (mg/dL)	102 ± 58	071 ± 35	0.017
Triglyceride-to-HDL cholesterol ratio	2.4 ± 1.4	1.2 ± 0.8	<0.001
Uric acid (mg/dL)	05.7 ± 0.8	05.1 ± 1.1	0.036
Glycaemic control			
Fasting glucose (mg/dL)	89 ± 6	85 ± 6	0.031
Fasting insulin (mU/l)	026 ± 16	10 ± 5	<0.001
Glycated haemoglobin (%)	05.3 ± 0.3	05.2 ± 0.2	0.064
HOMA-IR	05.7 ± 3.7	02.1 ± 1.2	<0.001
Endocrinology			
Leptin (µg/L)	046.9 ± 22.0	08.7 ± 6.3	<0.001

Data are expressed as mean ± SD. Abbreviations: LDL: low-density lipoprotein, HDL: High-density lipoprotein, HOMA-IR: Homeostatic Model Assessment of Insulin Resistance. Comparisons between two groups were performed using the independent-samples t-test or Mann–Whitney U test.

**Table 3:** Cardiopulmonary function during cardiopulmonary exercise testing in obese and lean subjects.

Peak	Obese (*n* = 29)	Lean (*n* = 29)	*p*-Value
Oxygen uptake (mL/min)	2070 ± 422	2219 ± 546	0.355
Oxygen uptake (mL/min/kg)	23.9 ± 4.8	40.8 ± 6.6	<0.001
Carbon dioxide output (mL/min)	2548 ± 557	2740 ± 714	0.265
Minute ventilation (L/min)	76 ± 19	83 ± 23	0.125
Tidal volume (L)	1.90 ± 0.46	1.90 ± 0.47	0.994
Breathing frequency (breaths/min)	41 ± 8	45 ± 9	0.108
Ventilatory equivalent O_2_	36.8 ± 4.7	37.7 ± 5.8	0.602
Ventilatory equivalent CO_2_	29.9 ± 2.8	30.5 ± 3.5	0.476
Respiratory exchange ratio	1.23 ± 0.07	1.23 ± 0.07	0.868
Oxygen pulse (mL O_2_/heart beat)	11.1 ± 2.0	11.9 ± 2.8	0.305
Work rate (W)	161 ± 35	189 ± 44	0.010
Oxygen uptake/Work rate (mL/min/W)	13.0 ± 1.3	11.7 ± 0.8	<0.001
Heart rate (bpm)	185 ± 11	186 ± 9	0.843
Mechanical efficiency (%)	21.3 ± 1.9	23.1 ± 1.4	<0.001
Net mechanical efficiency (%)	25.3 ± 2.2	27.1 ± 2.2	0.005
Lipid oxidation (g/min)	0.00 ± 0.00	0.00 ± 0.00	0.317
Carbohydrate oxidation (g/min)	4.74 ± 1.21	5.27 ± 1.60	0.167

Data are expressed as mean ± SD. Abbreviations: W: Watt, bpm: beats per minute. Comparisons between two groups were performed using the independent-samples t-test or Mann–Whitney U test.

## Data Availability

Data available on request due to restrictions (e.g. privacy or ethical).

## Supplementary Materials

The following are available online at https://www.mdpi.com/article/10.3390/ijerph182010578/s1, Table S1: Subject characteristics of obese and lean individuals, Table S2: Biochemical and hormonal parameters in obese and lean adolescents, Table S3: Cardiopulmonary function in rest, at VT1 and VT2 during cardiopulmonary exercise testing in obese and lean subjects, Figure S1: Sex differences of the oxygen uptake per work rate in relation to percentage of peak oxygen uptake in lean subjects and subjects with obesity during with maximal exercise testing, Figure S2: Sex differences of the mechanical efficiency.
