# Supplementary material for: Aberrant Mechanical Efficiency during Exercise Relates to Metabolic Health and Exercise Intolerance in Adolescents with Obesity
Source: Int J Environ Res Public Health. 2021 Oct 9;18(20):10578. doi: 10.3390/ijerph182010578 (PMC8535943; doi:10.3390/ijerph182010578)
Supplement: Supplementary file 1 [file ijerph-18-10578-s001.zip › ijerph-1349130-supplementary.pdf]

Aberrant Mechanical Efficiency during Exercise Relates to Metabolic Health and Exercise Intolerance in Adolescents with Obesity

Wouter M.A. Franssen, Guy Massa, Bert O. Eijnde, Paul Dendale, Dominique Hansen and Kenneth Verboven

Table S1. Subject characteristics of obese and lean individuals.

General features	Obese (n=29)		Lean (n=29)		p-value
	Male (n=15)	Female (n=14)	Male (n=16)	Female (n=13)	
Age (years)	13.9 ± 1.0	13.7 ± 1.2	14.0 ± 1.3	15.1 ± 1.6 [a]	0.023
Body weight (kg)	91.0 ± 18.0	84.9 ± 11.4	54.2 ± 12.6 [c,d]	55.4 ± 8.6 [a,b]	**<0.001**
Body height (cm)	169.2 ± 10.3	164.0 ± 6.0	167.6 ± 9.2	165.9 ± 8.9	0.433
Body height-SDS	0.82 ± 1.10	0.81 ± 0.89	0.61 ± 0.88	0.76 ± 1.19	0.930
BMI (kg/m^2)	31.7 ± 4.9	31.5 ± 3.4	19.0 ± 2.8 [c,d]	20.0 ± 1.8 [a,b]	**<0.001**
BMI-SDS	2.14 ± 0.38	2.07 ± 0.23	-0.26 ± 1.02 [c,d]	-0.04 ± 0.57 [a,b]	**<0.001**
Waist circumference (cm)	104.9 ± 13.7	101.0 ± 12.0	68.1 ± 7.1 [c,d]	66.6 ± 4.9 [a,b]	**<0.001**
Hip circumference (cm)	103.4 ± 9.7	104.8 ± 6.5	76.0 ± 9.3 [c,d]	82.0 ± 5.5 [a,b]	**<0.001**
Waist-to-hip ratio	1.01 ± 0.05	0.96 ± 0.08	0.91 ± 0.13 [d,e]	0.81 ± 0.04 [a,b]	**<0.001**
Body fat (%)	54.1 ± 9.3	40.6 ± 7.3 [f]	15.1 ± 5.9 [c,d]	21.5 ± 2.9 [a,b]	**<0.001**
Body fat (kg)	50.1 ± 16.5	34.8 ± 9.7 [f]	8.4 ± 4.6 [c,d]	12.1 ± 3.0 [a,b]	**<0.001**
Fat-free mass (kg)	40.9 ± 7.7	50.1 ± 7.2 [f]	45.7 ± 10.0	43.4 ± 6.1	**0.023**
Systolic BP (mmHg)	128 ± 14	121 ± 6	115 ± 10 [d]	113 ± 10 [b]	**<0.001**
Diastolic BP (mmHg)	77 ± 9	75 ± 10	69 ± 7	70 ± 8	0.069
Mean arterial pressure (mmHg)	94 ± 9	90 ± 7	84 ± 8 [d]	85 ± 8 [b]	**0.003**
Development stage					0.271
Tanner stage 1 (n)	3	0	1	1	
Tanner stage 2 (n)	0	0	2	0	
Tanner stage 3 (n)	6	2	4	0	
Tanner stage 4 (n)	2	1	6	1	
Tanner stage 5 (n)	4	11	3	11	

[A]: lean female vs. obese female, [B]: lean female vs. obese male, [C]: lean male vs. obese female, [D]: lean male vs. obese male, [E]: lean male vs. lean female, [F]: obese male vs obese female.

Table S2. Biochemical and hormonal parameters in obese and lean adolescents.

General features	Obese (n=29)		Lean (n=29)		p-value
	Male (n=15)	Female (n=14)	Male (n=16)	Female (n=13)	
**Cardiovascular risk factors**					
C-reactive protein (mg/l)	4.1 ± 6.1	4.3 ± 5.5	0.3 ± 0.4 [c,d]	0.6 ± 1.1 [b]	**<0.001**
Total cholesterol (mg/dl)	147 ± 28	169 ± 35	150 ± 22	149 ± 27	0.144
LDL cholesterol (mg/dl)	87 ± 26	101 ± 26	77 ± 20	72 ± 24 [a]	**0.012**
HDL cholesterol (mg/dl)	43 ± 12	47 ± 10	59 ± 15 [c,d]	63 ± 8 [a,b]	**<0.001**
Triglycerides (mg/dl)	103 ± 61	102 ± 58	72 ± 38	71 ± 31	0.133
Triglyceride-to-HDL cholesterol ratio	2.6 ± 1.8	2.1 ± 0.9	1.3 ± 0.9 [d]	1.1 ± 0.5 [b]	**0.003**
Uric acid (mg/dl)	5.9 ± 0.8	5.4 ± 0.8	5.5 ± 1.2 [f]	4.5 ± 0.6 [b]	**0.002**
**Glycaemic control**					
Fasting glucose (mg/dl)	91 ± 6	87 ± 5	85 ± 7	86 ± 6	0.071
Fasting insulin (mU/l)	29 ± 19	22 ± 12	8 ± 4 [c,d]	12 ± 6 [b]	**<0.001**
Glycated haemoglobin (%)	5.3 ± 0.3	5.4 ± 0.3	5.2 ± 0.3	5.2 ± 0.2	0.252
HOMA-IR	6.5 ± 4.4	4.8 ± 2.7	1.7 ± 0.8 [c,d]	2.6 ± 1.4 [a,b]	**<0.001**
**Endocrinology**					
Leptin (µg/l)	39.8 ± 19.6	54.1 ± 22.5	4.2 ± 1.9 [c,d]	14.2 ± 5.4 [a,b]	**<0.001**

[A]: lean female vs. obese female, [B]: lean female vs. obese male, [C]: lean male vs. obese female, [D]: lean male vs. obese male, [E]: lean male vs. lean female, [F]: obese male vs obese female.

Table S3. Cardiopulmonary function in rest, at VT1 and VT2 during cardiopulmonary exercise testing in obese and lean subjects.

	Obese (n = 29)	Lean (n = 29)	p-value
**Rest**			
Oxygen uptake (ml/min)	347 ± 84	312 ± 104	**0.036**
Oxygen uptake (ml/min/kg)	4.0 ± 0.9	5.9 ± 2.0	**<0.001**
Carbon dioxide output (ml/min)	310 ± 80	263 ± 90	**0.017**
Minute ventilation (l/min)	10 ± 3	9 ± 3	0.085
Tidal volume (l)	0.64 ± 0.26	0.63 ± 0.22	0.994
Breathing frequency (breaths/min)	17 ± 5	16 ± 5	0.347
Ventilatory equivalent O_2	30.5 ± 6.5	30.1 ± 5.4	0.864
Ventilatory equivalent CO_2	33.9 ± 3.7	35.5 ± 4.3	0.128
Respiratory exchange ratio	0.89 ± 0.11	0.85 ± 0.10	0.082
Oxygen pulse (ml O_2/heart beat)	3.9 ± 1.0	4.3 ± 1.7	0.203
Heart rate (bpm)	92 ± 12	74 ± 13	**<0.001**
Lipid oxidation (g/min)	0.04 ± 0.05	0.07 ± 0.04	**0.033**
Carbohydrate oxidation (g/min)	0.33 ± 0.16	0.19 ± 0.12	**<0.001**
**Ventilatory treshold 1**			
Oxygen uptake (ml/min)	1091 ± 185	1071 ± 276	0.468
Oxygen uptake (ml/min/kg)	12.7 ± 2.5	21.6 ± 3.5	**<0.001**
Carbon dioxide output (ml/min)	929 ± 234	958 ± 264	0.668
Minute ventilation (l/min)	27 ± 6	27 ± 6	0.975
Tidal volume (l)	1.01 ± 0.24	1.01 ± 0.25	0.992
Breathing frequency (breaths/min)	25 ± 5	23 ± 7	0.238
Ventilatory equivalent O_2	23.6 ± 3.0	23.1 ± 3.4	0.516
Ventilatory equivalent CO_2	26.7 ± 2.2	26.3 ± 2.7	0.523
Respiratory exchange ratio	0.88 ± 0.08	0.88 ± 0.09	0.726
Oxygen pulse (ml O_2/heart beat)	8.0 ± 1.6	9.0 ± 2.4	0.086
Work rate (W)	64 ± 16	77 ± 22	**0.020**
Oxygen uptake/Work rate (ml/min/W)	16.7 ± 4.1	14.5 ± 1.9	**0.009**
Heart rate (bpm)	131 ± 12	123 ± 11	**0.013**
Mechanical efficiency (%)	18.2 ± 2.7	20.5 ± 2.8	**0.002**
Net mechanical efficiency (%)	26.4 ± 5.5	29.2 ± 5.3	0.052
Lipid oxidation (g/min)	0.21 ± 0.13	0.22 ± 0.20	0.813
Carbohydrate oxidation (g/min)	0.78 ± 0.42	0.82 ± 0.55	0.732
**Ventilatory treshold 2**			
Oxygen uptake (ml/min)	1552 ± 358	1805 ± 496	0.068
Oxygen uptake (ml/min/kg)	18.6 ± 3.7	31.8 ± 5.1	**<0.001**
Carbon dioxide output (ml/min)	1824 ± 385	1860 ± 463	0.751
Minute ventilation (l/min)	51 ± 11	51 ± 10	0.889
Tidal volume (l)	1.48 ± 0.36	1.48 ± 0.37	0.996
Breathing frequency (breaths/min)	32 ± 7	32 ± 7	0.885
Ventilatory equivalent O_2	29.4 ± 3.0	28.5 ± 3.8	0.336
Ventilatory equivalent CO_2	26.7 ± 2.4	26.3 ± 2.5	0.590
Respiratory exchange ratio	1.10 ± 0.05	1.08 ± 0.08	0.222
Oxygen pulse (ml O_2/heart beat)	13.5 ± 1.3	12.5 ± 1.3	0.242
Work rate (W)	124 ± 28	138 ± 34	0.093
Oxygen uptake/Work rate (ml/min/W)	16.7 ± 4.1	14.5 ± 1.9	**0.009**
Heart rate (bpm)	167 ± 12	163 ± 14	0.244
Mechanical efficiency (%)	21.0 ± 1.9	22.7 ± 1.8	**<0.001**
Net mechanical efficiency (%)	26.1 ± 2.8	27.7 ± 2.5	**0.032**
Lipid oxidation (g/min)	0.00 ± 0.18	0.00 ± 0.24	0.446
Carbohydrate oxidation (g/min)	2.82 ± 0.68	3.12 ± 1.01	0.192

Data are expressed as mean ± SD. Abbreviations: W: Watt, bpm: beats per minute. Comparisons between two groups were performed using the independent-samples t-test or Mann-whitney U test.

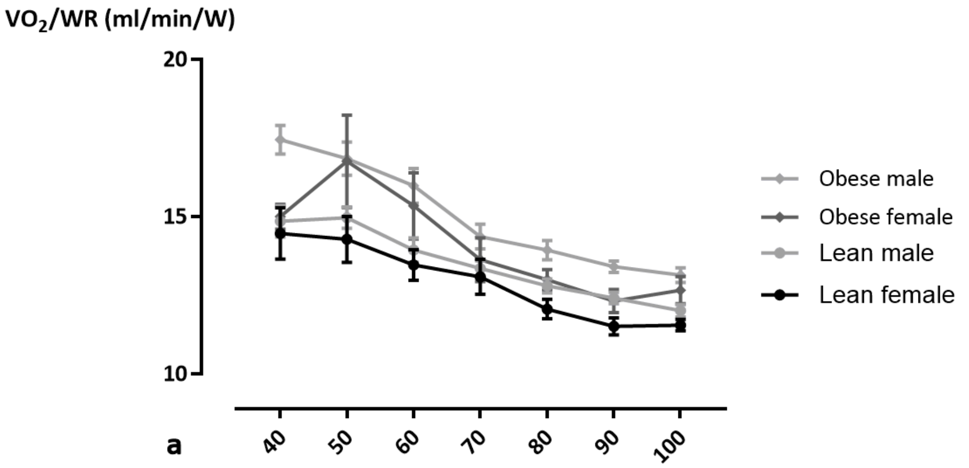

Figure S1. Sex differences of the oxygen uptake per work rate in relation to percentage of peak oxygen uptake in lean subjects and subjects with obesity during with maximal exercise testing. Data are presented as mean±SEM. Abbreviations: VO_2/WR: oxygen uptake per work rate, W: Watt.

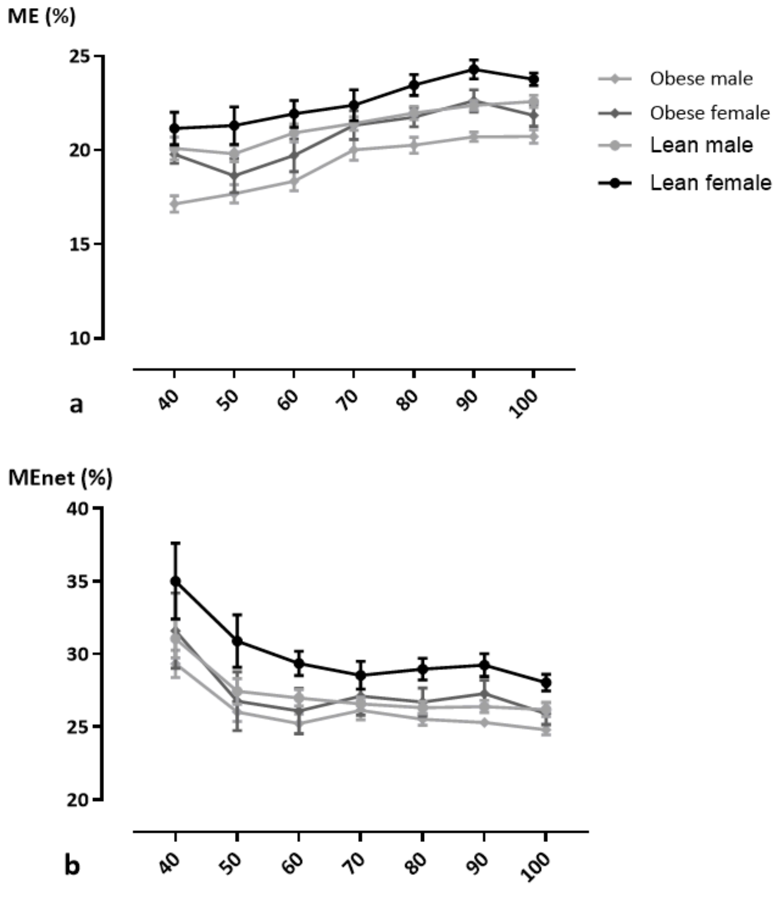

Figure S2. Sex differences of the mechanical efficiency (a) and net mechanical efficiency (b) in relation to percentage of peak oxygen uptake in lean subjects and subjects with obesity during maximal exercise testing. Data are presented as mean±SEM. Abbreviations: ME: mechanical efficiency, MEnet: net mechanical efficiency.
